# Measuring Structural Racism and Its Association with Racial Disparities in Firearm Homicide

**DOI:** 10.1007/s40615-022-01485-2

**Published:** 2022-12-12

**Authors:** Michael Siegel, Madeline Rieders, Hannah Rieders, Jinan Moumneh, Julia Asfour, Jinseo Oh, Seungjin Oh

**Affiliations:** Department of Public Health and Community Medicine, Tufts University School of Medicine, 636 Harrison Avenue, Boston, MA 02111 USA

**Keywords:** Racial health disparities, Structural racism, African Americans, Empirical measures

## Abstract

**Introduction:**

Structural racism is strongly related to racial health disparities. However, surprisingly few studies have developed empirical tools to measure structural racism. In addition, the few measures that have been employed have only considered structural racism at the neighborhood level. To expand upon previous studies, this paper uses a novel measure to measure structural racism at the county level for the non-Hispanic Black population.

**Methods:**

We used confirmatory factor analysis to create a model to measure the latent construct of structural racism for 1181 US counties. The model included five indicators across five dimensions: racial segregation, incarceration, educational attainment, employment, and economic status/wealth. Structural equation modeling and factor analysis were used to generate factor scores that weighted the indicators in order to produce the best model fit. The resulting factor scores represented the level of structural racism in each county. We demonstrated the utility of this measure by demonstrating its strong correlation with Black-White disparities in firearm homicide rates.

**Results:**

Our calculations revealed striking geographic differences across counties in the magnitude of structural racism, with the highest values generally being observed in the Midwest and Northeast. Structural racism was significantly associated with higher Black firearm homicide rates, lower White homicide rates, and a higher Black-White racial disparity in firearm homicide.

**Conclusions:**

These new measures can be utilized by researchers to relate structural racism to racial health disparities at the county level.

## Introduction

Structural racism has been defined as “the totality of ways in which societies foster racial discrimination through mutually reinforcing systems of housing, education, employment, earnings, benefits, credit, media, health care, and criminal justice [1, p. 1453].” The deep entrenchment of structural racism in the USA has been shown to cause adverse health consequences for people of color [1], and structural racism is recognized as a fundamental cause of racial inequities in health [2–4]. Methods to measure and quantify structural racism are essential in order to monitor and counteract its public health impact [5]. However, as Hardeman et al. point out, “empirical research has been slow to quantify structural racism and its impact on public health [6, p. 180],” and “the development of sound measures of structural racism is an urgent public health issue [6, p. 181].” There is no uniformly agreed upon strategy to measure structural racism [6], but approaches are rapidly evolving [5–17]. As stated by Groos et al., “there may not be a universal, single best or ‘gold standard’ operationalization of structural racism. However, valid, replicable and theoretically sound measures of structural racism are urgently needed in order to build evidence of its harms to population health and to identify pathways for intervention to advance racial health equity” [17, p. 191].

Our review of the literature on the measurement of structural racism suggests that there have been four “waves” in the evolution of these measurement approaches. The majority of studies that have measured structural racism (wave 1) have done so at the neighborhood level using a single component, such as residential racial segregation or neighborhood disadvantage in a socioeconomic indicator [5, 11, 16]. Dr. Alicia Riley has termed this approach “neighborhood effects research,” and she points out that studies of this nature date back to at least the 1970s [13]. The limitation of this approach is that it only accounts for one dimension of structural racism, while the definition we have accepted from Bailey et al. distinguishes at least nine dimensions [18]. As Hardeman et al. assert, measures of structural racism as determinants of health should ideally be multidimensional because “various forms of structural racism share the same pathway (for example, education inequity leads to employment inequity) or interact with one another and exert both their independent and joint effects to cause poor health among members of racial and ethnic minority groups [6, p. 183].” In addition, while structural racism has most often been measured at the neighborhood level, most of the policies that have institutionalized structural racism are adopted at levels beyond the individual neighborhood. Therefore, not only are novel measures of structural racism needed, but measures specific to larger geographic units—such as cities, counties, and states—are essential. As Hardeman et al. stated: “we encourage the use of a theory-driven approach in which appropriate geographic units are selected on the basis of the proposed underlying mechanisms of structural racism, suggested by prior research and theory” [6, pp. 182–183].

In the second wave of work to operationalize structural racism, researchers addressed this limitation by examining multiple dimensions of structural racism, while examining the impact of each dimension on health outcomes separately. At the same time, the unit of analysis tended to shift from the neighborhood to larger units such as counties or states. For example, Lukachko et al. examined structural racism at the state level by comparing the Black and White population in each state using 11 indicators across four domains: political participation (e.g., registered to vote), employment (e.g., labor force participation), educational attainment (bachelor’s degree or higher), and judicial treatment (e.g., incarceration rate) [19]. They found that for three of the indicators (state elected officials, employment, and incarceration), structural racism had a positive effect on the risk of myocardial infarction among Black people, but a negative effect on the risk of myocardial infarction among White people. Similarly, Wallace et al. measured structural racism at the state level using six indicators across the four domains of criminal justice, education, employment, and economic status, independently examining the relationship between each indicator and each state’s infant mortality rates [20]. They found that higher levels of racial inequity in unemployment led to a 5% increase in the Black infant mortality rate and that higher levels of racial inequity in educational attainment led to a 10% increase in the Black infant mortality rate. Bell et al. measured county-level structural racism using five indicators across three domains: education, employment, and economic status [21]. They reported that higher levels of racial inequities in poverty, unemployment, and homeownership in a county were associated with higher rates of obesity in that county. In another county level study, Chambers et al. independently examined the relationship between five indicators of structural racism across three domains—residential segregation, criminal justice, and political representation—and low birth weight and gestational age among Black and White women in California [22]. A major limitation of this line of research is that although multiple dimensions of structural racism are considered, the ways in which they may interact are not. In addition, they do not consider intersectionality, or the potential synergistic effects of the co-existence of multiple forms of structural racism.

A recent third wave of research aimed to address this limitation by developing indices or scales that combine indicators of structural racism across multiple domains and then examine the relationship between these overall structural racism indices and health outcomes. For example, Mesic et al. developed a structural racism index with five distinct indices applied at the state level [23]. The five dimensions of residential segregation, incarceration rates, educational attainment, economic indicators, and employment status were used to calculate an overall structural racism index for each state by averaging scores across the five domains [23]. The authors demonstrated the utility of this new state structural racism index by examining its relationship to Black-White disparities in firearm homicide rates at the state level in the USA [23]. Their study found a strong correlation between the state racism index and Black-White disparities in unarmed police shootings, with a 24% increase in the Black-White disparity ratio of unarmed police shooting rates for every 10-point increase in the overall state racism index [23]. Since then, this state structural racism index has also been applied to other racial health disparities, including COVID-19 mortality and vaccination rates [24, 25]. Specifically, Siegel et al. found that the highest Black-White disparity in COVID-19 mortality rates existed in five states with an average state structural racism index of 52.3 [24]. In contrast, the five states with the lowest disparity had an average state structural racism index of 40.8 [24]. Furthermore, Siegel et al. also employed the state structural racism index to better understand Black-White disparities in COVID-19 vaccination rates [25]. They found that the Black vaccination rate was 3.15% lower than the White vaccination rate for each one standard deviation increase in the Black state racism index [25]. In both studies, the state Black racism index was strongly correlated with Black-White racial disparities in the health outcomes.

At least one other research team has used the approach of summing data across multiple domains to construct a structural racism index. Homan et al. developed a state-level structural racism index by summing nine indicators covering five domains: segregation, political, judicial, educational, and economic [8]. The investigators demonstrated that high levels of structural racism on this index were associated with significantly lower levels of self-rated health among Black women. The primary limitation of this third approach is that it assumes all domains of structural racism are weighted equally since the indices are derived by simply averaging or summing across the domains. However, there is no conceptual or theoretical basis to support this assumption. Moreover, it is not entirely clear what the structural racism index actually represents since it is based on an arbitrary equal weighting of indicators across domains and of domains themselves. The equal weighting is arbitrary, rather than being based on either a conceptual theory or an empirical grounding that is present within the data. Finally, these indices are sensitive to measurement error.

In the most recent fourth wave of research, investigators are striving to avoid the limitations inherent in simply averaging or summing scores across dimensions of structural racism by developing methods to construct latent measures of structural racism across multiple dimensions in a way that retains the qualitative information provided by each dimension and is robust to measurement error [3, 9–12, 26, 27]. This latent construct approach allows modeling of both the independent and interactional effects of different dimensions [9, 10].

To date, two approaches have been advanced. First, Chantarat and colleagues at the Center for Antiracism Research for Health Equity at the University of Minnesota School of Public Health have developed a novel multidimensional measure of structural racism using latent class analysis [9, 10, 26]. In one study, they classified 2338 Public Use Microdata Areas into three classes with differing multidimensional structural racism profiles using latent class analysis [9]. They went on to demonstrate the utility of this approach by linking this new measure with COVID-19 vaccination rates across 55 community districts in New York City and demonstrating that the unique profiles of structural racism across these districts were associated with significant differences in vaccination rates. In a second study, Chantarat et al. used a similar approach and demonstrated that the multidimensional latent construct explained racial inequities in birth outcomes among Black and White women in Minnesota [10].

In the second approach, Dougherty and colleagues at the Johns Hopkins Bloomberg School of Public Health used confirmatory factor analysis to create a multidimensional latent construct for structural racism at the county level and showed that this construct was associated with higher body mass index among Black people and lower body mass index among White people [27]. Several advantages of this approach are that it minimizes the impact of measurement error, confirms the fit of the conceptual model rather than simply assuming (as in the third-wave studies) that a simple average is the best way to fit the data, and uses the data to organically derive appropriate weights for each of the structural racism dimensions rather than making arbitrary decisions. This approach was also used by, and further developed by, Brown and Homan [12]. Using confirmatory factor analysis, they modeled the latent construct of structural racism at the state level using nine indicators across five domains. They demonstrated that a single factor model achieved the best fit and then used factor analysis to generate factor scores for the latent construct of structural racism in each state. They then demonstrated a significant association between higher factor scores for a state and worse health outcomes among Black people, but not among White people, in that state.

In light of these new developments, Brown and Homan have noted that “further research is needed to assess the utility of using various types of latent measures of structural racism for studying racialized health inequalities [12, p. 445].” Dougherty and colleagues also stated the need for future studies to use their confirmatory factor analysis approach to explore the relationship between structural racism and other health outcomes (besides body mass index) [27]. This paper responds to these two calls.

In this study, we expand upon the previous research and improve upon our own [23–25] by using the confirmatory factor analysis approach to develop a latent construct of structural racism at the county level, confirm the model fit of this construct, identify the most appropriate indicators for each of five dimensions of structural racism by examining the model fit of each combination of potential indicators, and quantify that construct by generating factor scores resulting from the best fitting structural equation model using the weights that it gives to each indicator. We then test the utility of this new structural racism measure by examining its relationship with Black-White racial disparities in firearm homicide at the county level, while controlling for a host of potential confounding variables. Our underlying approach is guided by ecosocial theory, which hypothesizes that population health is influenced by higher level (macro-level) institutional and environmental discrimination that harms the health of Black people by limiting their access to resources necessary for advantage and disproportionately exposing them to risks that create disadvantage [28]. We are also guided by conflict theory and by race stratification theory which predicts that higher levels of structural racism would result in health benefits for White people and health harm for Black people [29–31]. For this reason, we chose, for our primary analysis, to model the impact of structural racism on racial disparities in firearm homicide, not on absolute rates of firearm homicide among either the Black or White population in each county. Our hypothesis is that structural racism simultaneously creates White advantage and Black disadvantage, thus resulting in a strong predictive value of structural racism in explaining racial disparities in health outcomes. We test this hypothesis directly by comparing the extent to which structural racism explains the variation in White firearm homicide rates, Black firearm homicide rates, and the ratio of Black to White firearm homicide rates across counties.

Our three major research questions are as follows:

1. Can a latent variable be constructed to represent the construct of structural racism at the county level using indicators across multiple dimensions while minimizing the impact of measurement error and confirming a good model fit?
2. Do the factor scores resulting from that model predict Black-White racial disparities in firearm homicide rates across counties?
3. Does structural racism predict the racial disparity in firearm homicide across counties better than it predicts absolute rates of race-specific firearm homicide?

To the best of our knowledge, this is the first paper to explicitly model the relationship between structural racism based on a confirmatory factor analysis approach and racial disparities in a health outcome; in this case, firearm homicide rates.

## Methods

### Design Overview

Using confirmatory factor analysis, we developed a structural equation model to represent the latent construct of structural racism at the county level using a total of five indicators across five domains: (1) residential segregation; (2) incarceration; (3) employment; (4) economic status/wealth; and (5) education. A number of possible indicators for each domain were tested and we settled upon the model which produced the best model fit statistics. Factor scores for each county were generated and served as estimates of the level of structural racism in each county. To test the utility of this measure, we examined the relationship between the factor scores for each county and that county’s observed ratio of non-Hispanic Black firearm homicide rate to non-Hispanic White firearm homicide rate over the period 1999–2020, while controlling for a wide range of potential confounding variables. We separately modeled the relationship between structural racism and the absolute White firearm homicide rate, the absolute Black firearm homicide rate, and the ratio of Black to white firearm homicide rates (i.e., the magnitude of the racial disparity in firearm homicide in that county). All data used in the factor analysis were for the year 2020, with the exception of incarceration data which were from the year 2010. Data on the structural racism indicators were derived from the United States Census Bureau, using the 2020 Decennial Census and the 2020 American Community Survey 5-year estimates.

### Structural Racism Measures and Data Sources

We chose the domains to measure structural racism based on Bailey et al.’s [18] definition of structural racism, an examination of domains used by other researchers, and consideration of data availability. The domains outlined by Bailey et al. are education, employment, housing, criminal justice, benefits, credit, earnings, media, and health care [18]. Chantarat et al. measured structural racism using six domains: residential segregation and inequality in education, employment, income, homeownership, and criminal justice [9, 10]. Dougherty used education, housing, employment, criminal justice, and health care [27]. Mesic et al. used segregation, education, employment, economic status, and incarceration [23]. Homan et al. used segregation and political, judicial, educational, and economic inequalities (13). Lukachko et al. used political participation, employment, educational attainment, and incarceration [19].

Because the major purpose of our measuring structural racism is to correlate it with health outcomes, we opted not to include the domain of health care inequities because these would be expected to directly affect health outcomes and would therefore be a direct part of the causal pathway. Beyond that, we attempted to include all other identified domains where race-specific data were available at the county level for a large number of counties and to attempt to use indicators used in prior research. At the county level, we could not find adequate data availability for measurement of race-specific political participation or for media exposure. We were able to find a measure that in a way combines benefits, credit, and earnings, as it measures the expected 10-year trajectory of Black compared to non-Hispanic White children in a given income stratum today, predicting the income stratum that the youth will be in after 10 years, based on historical data at the county level. These data, termed the Racial Opportunity Gap, were kindly provided by Dr. Rourke O’Brien at Yale University [32]. The racial opportunity gap is the difference in economic mobility outcomes of Black vs. White children, taken as the difference in the average national income percentile ranking in adulthood achieved between White and Black individuals in the same county born to parents at the 25th percentile of the national income distribution [32]. Because it directly measures economic mobility, it is an ideal measure based on our conceptual hypotheses regarding the impact of structural racism on wealth and economic mobility. We were able to create a robust set of indicators for the economic status/wealth domain by combining the racial opportunity gap with data on income, homeownership, labor force participation, and poverty.

As a result of these considerations, we decided upon the use of five domains and 15 possible indicators across these domains (Table 1): (1) residential segregation (the index of dissimilarity, isolation index, entropy index, and Index of Concentration at the Extremes for racialized economic segregation); (2) education (Black-to-non-Hispanic White ratio of persons without a college degree and of persons without a high school degree); (3) employment (Black-to-non-Hispanic White ratio of persons in service occupations, non-Hispanic White-to-Black ratio of persons in managerial occupations, and Black-to-non-Hispanic White unemployment rate ratio; (4) economic status and wealth (Black-to-non-Hispanic White ratios of poverty, proportion living in rental housing, and labor non-participation rate, non-Hispanic White-to-Black ratio of median household income, and racial opportunity gap); and (5) incarceration (Black-to-non-Hispanic White ratio of the proportion of people detained or in local jails). The reason we used the White-to-Black ratios for median income and percentage of workers in managerial occupations is to ensure that higher ratios were indicative of structural racism in order to be consistent and avoid interpretation errors. Table 1 defines each indicator and provides the specific data source and years of data used.

**Table 1:** Dimensions, indicators, definitions, and data sources for development of the County Black Structural Racism Index

Dimension	Indicator	Definition	Data source
Incarceration	Detention or jailing ratio	Ratio of the Black to White local jailing rate. Local jailing rate is the number of persons in local jails or detention centers per 100,000 population	United States Census Bureau, Decennial Census, 2010
Segregation	Index of dissimilarity	Represents the percentage of Black people who would have to move in order to achieve an equal distribution of White and Black people across all blocks within a geographic area	United States Census Bureau, Decennial Census, 2020
	Isolation index	It can be interpreted as the extent to which Black members of a block are exposed only to one another	
	Entropy index	Measures the spatial distribution of Black and White people within a county	
	Index of concentration of extremes (ICE) for racialized economic segregation	Measures the difference between the number of White people living at over 80th percentile of income and the number of Black people living at below the 20th percentile of income in 2020	
Economic status/wealth	Income ratio	Ratio of median household income for the White population to median household income for the Black population	United States Census Bureau, American Community Survey’s 5-year estimates for 2020
	Rental ratio	Ratio of the proportion of Black people in rental housing to that of White people in rental housing	
	Poverty ratio	Ratio of the Black poverty rate to the White poverty rate	
	Non-labor participation ratio	Ratio of the Black labor non-participation rate to the White labor non-participation rate. Labor non-participation means not participating in the labor force	
	Racial opportunity gap	Difference in economic mobility of Black vs. White children, taken as the difference in the average national income percentile ranking in adulthood achieved between White and Black individuals in the same county born to parents at the 25th percentile of the national income distribution	Data provided by Dr. Rourke O’Brien at Yale University; from Opportunity Atlas
Education	No high school ratio	Ratio of the proportion of Black people without a high school degree to the proportion of White people without a high school degree	United States Census Bureau, American Community Survey’s 5-year estimates for 2020
	No college ratio	Ratio of the proportion of Black people without a bachelor’s degree to the proportion of White people without a bachelor’s degree	
Employment	Unemployment ratio	Ratio of the Black unemployment rate to the White unemployment rate	United States Census Bureau, American Community Survey’s 5-year estimates for 2020
	Managerial occupation ratio	White to Black ratio of proportion of workers in managerial occupations	
	Service occupation ratio	Black to White ratio of proportion of workers in service occupations	

We used 2020 data for all indicators with the exception of the jailing rates, which were obtained from the 2010 Decennial Census and have not yet been released for 2020. For most indicators, the U.S. Census Bureau does not provide estimates for the non-Hispanic Black population so we used data for “All Black” (defined as anyone of single race who identified as Black). The “White” population consisted of Census estimates of the non-Hispanic White population.

Following Dougherty et al. [27], we used confirmatory factor analysis to (1) identify the combination of indicators within each domain that produced the best model fit; and (2) identify the relative weighting of each indicator in the composite measure. Also following Dougherty et al. [27], we developed structural equation models containing every combination of indicators but requiring that there be at least one indicator present for each domain. All models were unifactorial. We fit the models with maximum likelihood estimation after first standardizing each indicator so that the relative weightings could be compared without attention to the scale of each item. We used robust standard errors which produce unbiased estimates even in the presence of skewed data. Our models allowed for correlation between error terms for the indicator variables and we chose the final covariance structure based on goodness of fit criteria. The four criteria used to assess model fit were (1) root mean square error of approximation, (2) Tucker-Lewis index, (3) confirmatory fit index, and (4) the standardized root mean square residual. In the factor analyses, we included only counties with a Black population of at least 1000 to avoid using unstable estimates of parameters for the Black population. The factor scores were standardized so that for any given county, the score represents the number of standard deviations that the county is away from the mean for all counties. All analyses were conducted using the *sem* and *factor* procedures in STATA version 17.

### Structural Racism Final Model

The model with the best fit that emerged from our structural equation modeling and factor analysis was a single factor model that contained five indicators—one from each domain—and included three correlated error terms. The fit statistics for the final model were excellent, with a root mean square error of approximation of 0.021, a confirmatory fit index of 0.996, a Tucker-Lewis fit index of 0.980, and a standardized root mean square residual of 0.012. All factor loadings were statistically significant.

**Fig. 1:**
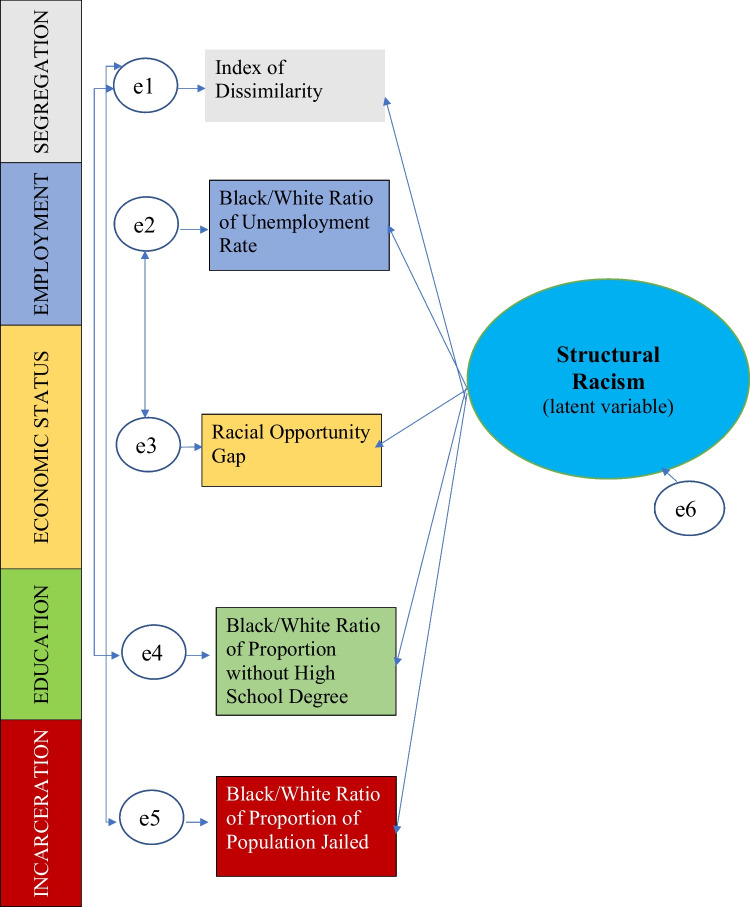
Diagram of final structural equation model. Oval shape indicates the latent variable of structural racism. Rectangular boxes represent the exogenous variables, in this case, indicators covering each of the five domains of structural racism considered in this model

The structural equation model is shown as Fig. 1. The indicators in each domain were as follows.

#### ***Incarceration***

The incarceration dimension included one indicator: the Black-to-White ratio of the local detention or jailing rate in the county. The number of people jailed or detained in a given county was obtained from the 2010 Decennial Census because group quarters data from the 2020 Decennial Census has not yet been released. This indicator could not be calculated for counties that do not have a local jail or detention center. Those counties were not included in the analysis. We were able to calculate jailing/detention ratios for a total of 2362 counties (data were missing for 787 counties).

#### Segregation

The segregation dimension included one indicator: the index of dissimilarity, a measure of the differential distribution of two population groups [33]. It was calculated at the block level using data collected from the 2020 Decennial Census and is in a range from 0 to 100, with higher values indicating higher levels of segregation.

#### Economic Status/Wealth

The economic status dimension included one indicator: the racial opportunity gap. The racial opportunity gap was introduced as an economic measure of structural racism by O’Brien et al. in a 2020 article [32]. It is described by the authors as follows: “The racial opportunity gap captures the difference in the adult earnings of black and white children born to families at the same income level. Specifically, the opportunity gap measures the difference in the income percentile ranking in adulthood between black and white children who started at the same place in the national income distribution [32, p. 2].” County-level data on the racial opportunity gap were kindly provided by O’Brien [32].

#### Education

The education dimension included one indicator: the Black to White ratio of the proportion of adults ages 25 + without a high school degree. Data were obtained from the American Community Survey 5-year estimates for 2020.

#### Employment

The employment dimension included one indicator: the Black-to-White ratio of the unemployment rate. Data were obtained from the American Community Survey 5-year estimates for 2020.

#### Structural Racism Factor Scores

Using the weighting of indicators suggested by the final model, we generated standardized factor scores for each county which represent the level of the latent structural racism construct for that county in terms of the number of standard deviations; its factor score is from the mean for all counties (which was set at zero). Based on data availability, we were able to generate factor scores for 1181 counties. These counties accounted for 82% of the total US population and 89% of the non-Hispanic Black US population.

#### Firearm Homicide Measures and Data Sources

Using the CDC WISQARS database [34], we obtained the county-level, age-adjusted firearm homicide rates for the non-Hispanic Black and non-Hispanic White populations for the period 2016–2020. We divided the Black firearm homicide rate for each county by the White firearm homicide rate to calculate the racial disparity (defined as the ratio of Black to White age-adjusted firearm homicide rates). Because WISQARS only reports cells that have at least 10 deaths, we were unable to obtain data for some counties; thus, our analysis is based on a total of 395 counties.

### Data Analysis

Because of the skewed distribution of homicide rates, we log-transformed these rates as well as the rate ratio. Thus, the outcome variables were the logged ratio of the Black to White firearm homicide rate in each county as well as the log of the absolute Black and White firearm homicide rates in each county. The main predictor variable was the structural racism factor score for that county. We controlled for the following variables which are known to be strongly related to firearm homicide rates: total population, population density, proportion of the population without a high school degree, unemployment rate, labor non-participation rate, poverty rate, median household income, percent of the population living in rental housing, percentage of young adults (ages 15–29), percentage male, and the age-adjusted non-firearm homicide rate. The exponentiated regression coefficients (multiplied by 100) indicate the percentage change in the outcome variable for each one standard deviation increase in the structural racism factor score.

## Results

### Descriptive Results

**Fig. 2:**
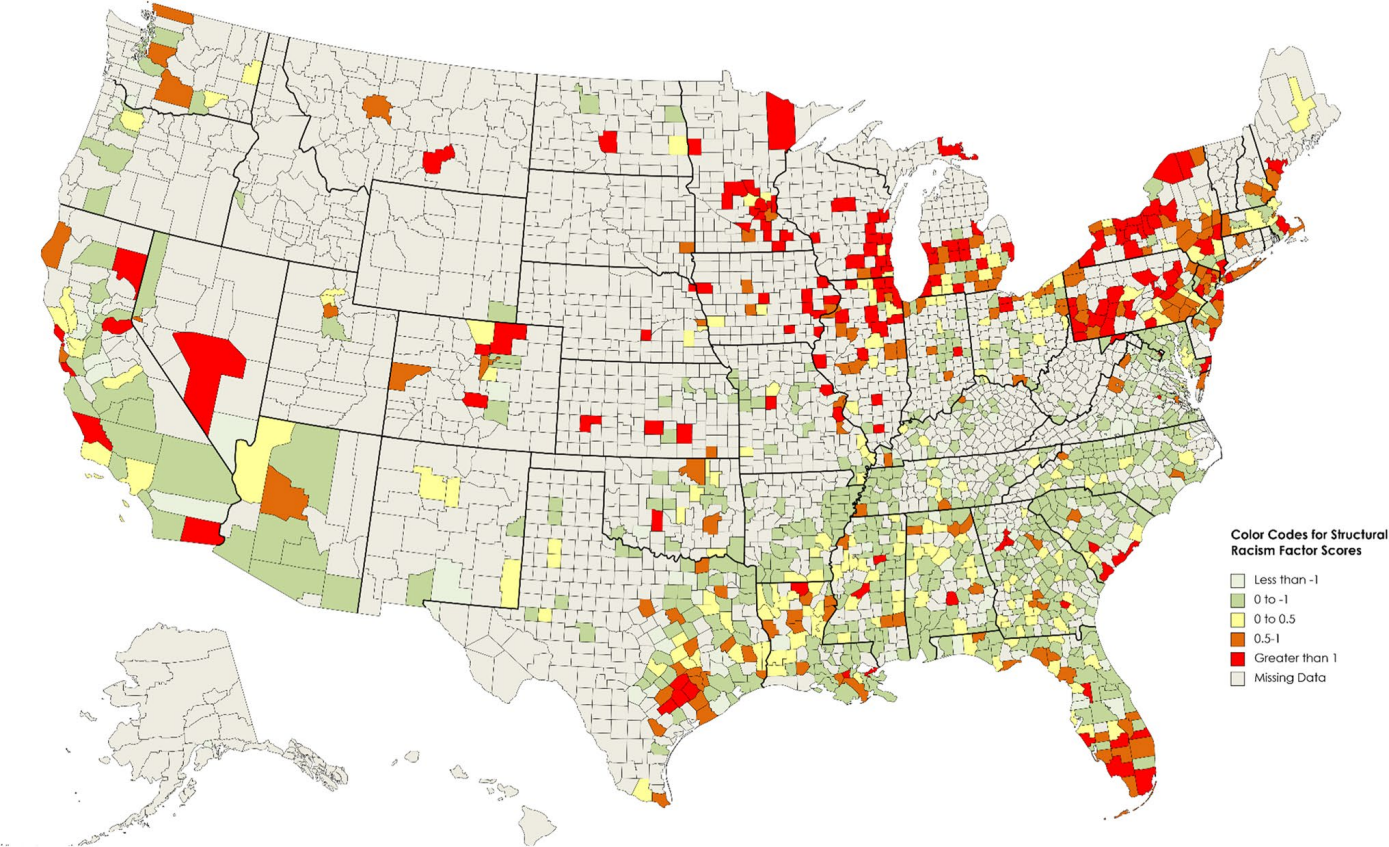
Heat map showing the magnitude of the standardized structural racism factor scores for US counties (*N* = 1181)

Among all 1181 counties, the standardized structural racism factor scores ranged from a low of -2.52 to a high of + 5.29. A color-coded US map displays the standardized structural racism factor scores for each of the 1181 counties for which we were able to generate such scores (Fig. 2). Table 2 displays the indicators and standardized structural racism factor scores for counties with the 25 highest and 25 lowest scores in the USA. The three counties with the highest standardized structural racism factor scores were Rice County, Minnesota (+ 5.29); Sherburne County, Minnesota (+ 4.77); and Henry County, Illinois (+ 4.75). All three had high levels of racial segregation with indices of dissimilarity above 73 and high levels of racial disparity in jailing rate (ratios above 30). Strikingly, 18 of the 25 counties were located in the upper Midwest, and 16 were located in four Midwest states (Illinois, Wisconsin, Michigan, and Minnesota).

**Table 2:** Summary of county structural racism indicators and factor scores for the top 25 and bottom 25 counties in terms of their factor scores

County, state	Index of dissimilarity	No high school ratio	Unemployment ratio	Racial opportunity gap	Local jailing rate ratio	Structural racism factor score
Top 25 counties						
Rice County, Minnesota	74.35	10.07	2.14	0.10	30.72	5.29
Sherburne County, Minnesota	73.21	4.80	2.22	0.10	39.50	4.77
Henry County, Illinois	83.47	5.70	5.49	0.12	30.74	4.75
New York County, New York	70.83	10.60	2.60	0.18	29.09	4.65
Butler County, Kansas	78.29	4.43	0.12	0.15	42.94	4.55
Nicollet County, Minnesota	67.64	8.47	3.50	0.17	29.93	4.42
Williamson County, Illinois	72.74	0.87	1.23	0.10	56.22	4.17
Kandiyohi County, Minnesota	81.14	7.47	4.93	0.28	9.27	3.83
Fayette County, Texas	75.29	4.85	4.35	0.17	20.26	3.35
Ulster County, New York	60.15	2.66	0.92	0.10	47.52	3.17
Ozaukee County, Wisconsin	64.77	7.64	1.69	0.03	28.05	3.15
Muscatine County, Iowa	74.04	0.53	1.95	0.20	33.96	3.12
Olmsted County, Minnesota	62.26	10.15	4.13	0.16	8.76	2.92
Benton County, Minnesota	76.93	5.07	0.50	0.20	16.79	2.90
Outagamie County, Wisconsin	70.35	3.30	3.66	0.16	30.41	2.89
Marathon County, Wisconsin	81.26	2.15	1.32	0.11	27.82	2.88
Clinton County, Michigan	72.04	2.83	0.34	0.11	32.08	2.87
Hennepin County, Minnesota	64.64	9.17	2.96	0.15	13.32	2.87
Hall County, Nebraska	78.67	6.41	12.02	0.08	12.32	2.83
Stearns County, Minnesota	76.93	5.71	5.81	0.23	9.96	2.78
Arlington County, Virginia	54.55	11.88	1.59	0.10	14.57	2.76
Hunterdon County, New Jersey	68.76	3.56	1.70	0.15	31.84	2.75
Waukesha County, Wisconsin	69.04	5.16	2.10	0.10	24.26	2.72
Dubuque County, Iowa	71.08	1.70	8.66	0.20	26.76	2.57
Bay County, Michigan	73.25	2.78	3.78	0.15	22.24	2.33
Bottom 25 counties						
Gloucester County, Virginia	37.58	1.31	0.18	0.06	2.77	− 2.52
Hoke County, North Carolina	37.36	1.38	0.92	0.07	4.16	− 2.38
Liberty County, Georgia	41.02	0.83	1.49	0.07	3.67	− 2.38
Columbia County, Georgia	39.78	0.81	1.85	0.10	3.19	− 2.36
Pulaski County, Missouri	45.52	0.34	2.97	0.05	2.64	− 2.25
Montgomery County, Virginia	45.50	0.70	0.63	0.06	2.04	− 2.18
Comanche County, Oklahoma	43.80	0.87	2.63	0.08	2.54	− 2.12
Harrisonburg city, Virginia	37.20	1.97	1.71	0.11	4.18	− 2.07
Prince George County, Virginia	39.03	1.43	1.61	0.10	3.10	− 2.06
Geary County, Kansas	38.30	2.22	0.35	0.08	3.56	− 2.03
Montgomery County, Tennessee	41.97	1.27	1.74	0.09	3.71	− 2.03
Stafford County, Virginia	43.77	1.05	1.42	0.08	2.33	− 2.01
Lee County, Georgia	42.32	1.67	3.00	0.08	2.94	− 2.00
Coryell County, Texas	44.10	1.78	1.09	0.08	1.84	− 1.93
Campbell County, Virginia	44.23	1.16	1.62	0.10	4.31	− 1.92
Caroline County, Virginia	41.19	2.64	0.62	0.04	3.67	− 1.91
Rutherford County, Tennessee	43.89	1.26	2.00	0.08	2.81	− 1.89
Clayton County, Georgia	50.30	0.59	1.12	0.08	0.84	− 1.88
Cumberland County, North Carolina	45.87	1.29	1.54	0.08	3.20	− 1.88
Douglas County, Georgia	49.73	0.62	1.14	0.08	1.70	− 1.85
Flagler County, Florida	43.40	1.48	0.38	0.09	3.65	− 1.84
Lake County, Tennessee	50.02	1.20	2.53	0.08	1.25	− 1.82
Petersburg city, Virginia	48.11	1.39	3.32	0.05	3.33	− 1.81
Onslow County, North Carolina	43.30	2.00	1.32	0.07	4.34	− 1.80
Rockdale County, Georgia	49.93	0.68	1.38	0.12	1.50	− 1.75

The three counties with the lowest structural racism factor scores were Gloucester County, Virginia (− 2.52); Hoke County, North Carolina (− 2.38); and Liberty County, Georgia (− 2.38). All three had relatively low levels of racial segregation with indices of dissimilarity below 42 and low racial opportunity gaps (0.06–0.07). Twenty-one of the 25 counties were located in the Southeast, with 20 of them representing just four states (North Carolina, Virginia, Tennessee, and Georgia).

### Analytic Results

**Fig. 3:**
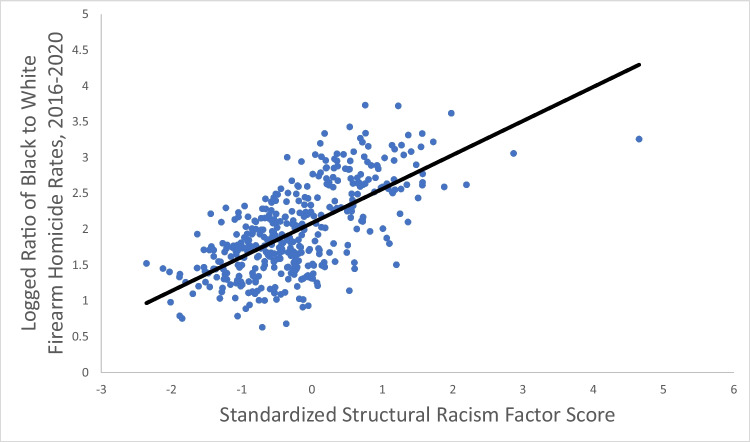
Correlation between the 2020 county structural racism factor scores and the logged ratio of county-level, 2016–2020 age-adjusted Black to White firearm homicide rates (*N* = 395 counties)

At the county level, the correlation between the structural racism factor score and the log of the Black-White racial disparity in firearm homicide was 0.66 (Fig. 3), indicating that 43% of the variation in the difference in magnitude in racial disparity in firearm homicide rates across states was explained by the single factor of the county structural racism factor score. In a bivariate linear regression analysis, the structural racism factor score was significantly associated with the racial disparity in firearm homicide, with each one standard deviation increase in the factor score increasing the firearm homicide rate ratio by a factor of 1.60 (95% confidence interval, 1.52–1.70) (Table 3). This relationship was unaltered by the inclusion of the county-level control variables. The structural racism factor score was significantly associated with an increase in the Black firearm homicide rate and a decrease in the White firearm homicide rate. While the structural racism factor score explained only a small amount of the variation in the Black homicide rate (6%) or White homicide rate (19%), it explained a large amount of the variation in the racial disparity (43%).

**Table 3:** Results of linear regression analyses modeling relationship between structural racism factor scores and racial disparity between non-Hispanic Black and non-Hispanic White age-adjusted firearm homicide rates, 2016–2020 (*N* = 395 counties)

Outcome variable	Regressors	Exponentiated regression coefficient for standardized structural racism factor score	95% confidence interval	*p*-value	*R*^2^
Log of Black firearm homicide rate	Bivariate	1.16	1.09–1.24	< 0.001	0.06
	Multivariate*	1.25	1.15–1.34	< 0.001	0.52
Log of White firearm homicide rate	Bivariate	0.72	0.68–0.77	< 0.001	0.19
	Multivariate*	0.72	0.67–0.76	< 0.001	0.76
Log of ratio of Black to White firearm homicide rates	Bivariate	1.60	1.52–1.70	< 0.001	0.43
	Multivariate*	1.74	1.60–1.89	< 0.001	0.55

^*^These regressions control for total population, population density, proportion of the population without a high school degree, unemployment rate, labor non-participation rate, poverty rate, median household income, percent of the population living in rental housing, percentage of young adults (ages 15–29), percentage male, and the age-adjusted non-firearm homicide rate. The exponentiated regression coefficients (multiplied by 100) indicate the percentage change in the outcome variable for each one standard deviation increase in the structural racism factor score

## Discussion

To our knowledge, this is just the second paper to use confirmatory factor analysis to create a single, composite measure of structural racism at the county level and the first to use this measure to explicitly model the racial disparity in a health outcome. Using publicly available data sources, we were able to derive structural racism factor scores for 1181 counties, accounting for 82% of the US population and 89% of the non-Hispanic Black population. We found that the structural racism factor scores were associated with significantly increased Black firearm homicide rates, significantly lower White firearm homicide rates, and significantly greater Black-White racial disparities in firearm homicide. We believe this is the first study to report a relationship between a multidimensional structural racism measure and the Black-White racial disparity in firearm homicide, although an earlier study reported a strong relationship between residential segregation and the Black-White firearm homicide rate disparity at the state level [35].

While most previous measures of structural racism have focused on single variables at the neighborhood level, this paper derives a composite structural racism index that incorporates five different domains and extends the geographic scope to the county level. The extension of structural racism measures to higher levels of geography is beneficial because the policies that have institutionalized structural racism have most often been implemented at these levels, and it is government bodies at each of these levels that have the power to enact policies to create change. Our research builds upon the work of Chantarat et al. [9, 10], Brown and Homan [12], and Dougherty et al. [27], who paved the way for this work by developing approaches to model the intersectional and interactive nature of the multiple dimensions of structural racism using techniques that minimize the impact of measurement error in the indicator variables and that do not rely on arbitrary assumptions about the weighting of these indicator variables. In our own prior work, we merely averaged scores across each dimension to derive a composite structural racism index [23–25], which assumes that each dimension contributes equally to the latent construct of structural racism. Here, we used structural equation modeling and factor analysis to empirically derive weightings and we then ensured that the final model adequately fit the data.

Our finding that structural racism appears to be associated with higher Black firearm homicide rates and lower White firearm homicide rates is consistent with conflict theory and race stratification theory which predict that higher levels of structural racism would result in health benefits for White people and health harm for Black people [29–31]. We found that each one standard deviation increase in the structural racism factor score in a county is associated with a 16% increase in the Black firearm homicide rate in that county and a 28% decrease in the White firearm homicide rate in that county. Brown and Homan similarly found that structural racism decreased self-rated mental health among Black people while increasing it among White people [33]. However, it should be noted that for some health outcomes, structural racism has been found to affect only Black health outcomes, not those for the White population [19, 33]. This could be related to the phenomenon, described in detail by Metzl [36], that in perpetuating White hegemony, white-controlled governments often sacrificed the well-being of White community members in order to avoid having to do anything to provide equal treatment to the Black population. For example, rather than integrating schools, many Southern states simply canceled school for all children, Black and White [36].

We believe that this is one of the first papers to explicitly model racial disparities in a health outcome as the dependent variable, rather than absolute levels of that outcome. We find, in fact, that the structural racism factor scores explain a far greater proportion of the variation in the racial disparity in firearm homicide than of the variation in either White or Black firearm homicide rates. This structural racism measure helps explain why different counties have different levels of racial disparity in firearm homicide, and it explains that racial disparity better than the absolute rates of race-specific firearm homicide observed in those counties.

One notable finding from this research is that the highest levels of structural racism tended to be in the Midwest and Northeast, while levels in the South and West were generally lower. This may be surprising given that many people associate the South with slavery and racism and may therefore assume that structural racism is highest in this region. However, structural racism was not limited to the institution of slavery. In fact, after the abolition of slavery, structural racist practices were widely implemented in order to maintain White hegemony despite the newly found freedom of Black slaves [37].

Structural racism is multidimensional and includes many factors beyond the history of slavery. Many structural racist policies were implemented in response to the abolition of slavery and the migration of many former slaves and their descendants to the Midwest and East [37]. These policies include redlining, sundown towns, discriminatory lending practices, restrictive covenants, limitation of policy benefits (such as the GI Bill) to White people, employment discrimination, unequal distribution of educational resources and opportunities, racist policing practices and criminal justice policy, mass incarceration, and racial discrimination in health care access and quality of care [1, 37, 38]. Since freed slaves tended to stay on plantations to work as sharecroppers, there was ironically geographical integration in the South, in contrast to the Midwest and Northeast, whose Black populations consisted largely of former slaves and their descendants who migrated during the twentieth century and who could be easily segregated by racist housing, zoning, and lending practices. The comparatively lower structural racism indices observed in the South are not due to progressive policies, but rather to the legacy of enslavement and post-slavery labor exploitation of Black people on White-owned property and to the fact that one of the ways that Southern policy makers avoided the equal treatment of Black people was to deny resources and rights, including public education, social services, and voting rights to all poor people, whether White or Black [36]. Thus, levels of educational attainment, employment, and economic status tend to be lower for the White population in the South, which ironically lowers the differential between the Black and White populations.

It may be helpful to provide profiles of the top three counties in terms of the structural racism factor scores. Where possible, we have attempted to provide some historical context.

Rice County, Minnesota, had approximately 67,000 residents in 2020, of whom 4353 (6.5%) identified as non-Hispanic Black. Perhaps the most striking indicators we observed for Rice County were its astounding racial disparities in educational attainment and poverty. While only 4.1% of White residents of the county do not have a high school degree, the proportion for the Black, non-Hispanic population is 41.2%, a tenfold disparity. This makes Rice County the 168th lowest rate of high school attainment for the Black population out of 2590 counties in the USA for which we could obtain such data. Furthermore, only 8% of Black people in Rice County have a 4-year college degree. The Black poverty rate is 39.6% compared to 6.9% among the non-Hispanic White population. The jailing rate for Black people in Rice County is 30 times higher than that for White people. According to the Prison Policy Initiative, an astounding 53% of Black residents of Rice County were incarcerated in 2000 [39]. While Black people comprised only 1.3% of the population of Sherburne County in 2000, they comprised 34% of the incarcerated population of the county [39]. According to the county’s public health department: “Over the past 20 years, Rice County has experienced a significant racial demographic change due to an increase in immigrants and refugees calling the area home. According to the U.S. Census Bureau, in the past two decades the county has gone from 93% White to 84% White and is now one of the more diverse counties in Minnesota. Notably, the Black/African-American population has increased 308% since 2000. The influx of new residents, many of whom arrive with large families, has led to a dramatic increase in housing demand. For example, according to the Rice County Housing and Redevelopment Authority, 1% of Minnesota Sect. 8 households have families with 10 or more members; in Rice County, that figure is 6%. Compared with nearby counties, Rice County has more unskilled jobs in food processing, warehousing, construction, and other industries, creating a draw to the area, but the county doesn’t have enough housing for the size of the families moving in. Lack of affordable housing is a pressing issue — and one of the leading social determinants of health in Rice County [40].” According to a ProPublica analysis of statistics from the Northfield Public School District in Rice County for the 2014–2015 school year, Black students were 4.8 times more likely to be suspended than White students [41]. In the Faribault Public School District, Black students were 2.2 times more likely to be suspended than White students and academically were an average of 3.4 grades behind White students [41]. Although Black students made up 19% of the district enrollment, not a single Black student was enrolled in an Advanced Placement course, and only 3% were enrolled in Gifted and Talented programs [41]. As recently as 2020, the Northfield superintendent of schools reported that there were numerous incidents involving public displays of racism, saying that: “there have been numerous incidents of racism right here, our beloved Bridgewater Principal Nancy Antoine, in the last 18 months, has received two horrific cards that called her the ‘N’ word and told her to go back to a continent which she’s never lived. What’s right about that? There’s nothing right about that. Over winter break I got a call that I had to go to the Middle School to take a look. Someone took a tremendous amount of time to sketch out a white power symbol in the snow outside the Middle School” [42]. As recently as 2017, a sergeant in the Rice County Sheriff’s Department created a public controversy when he issued a racist Tweet in response to the Philando Castile settlement [43].

Sherburne County, Minnesota, had approximately 97,183 residents in 2020, of whom 3648 (3.8%) identified as non-Hispanic Black. Perhaps the most striking indicators we observed for Sherburne County were its astounding racial disparities in poverty rate and homeownership. The White poverty rate is only 3.6%, while the Black poverty rate is 25.6%, a sevenfold disparity. While only 15% of White residents of Sherburne County live in rental housing, 81% of Black residents do, more than a fivefold disparity. There is also a 40-fold disparity in the jailing rate. According to the Prison Policy Initiative, an astounding 55% of Black residents of Sherburne County were incarcerated in 2000 [39]. While Black people comprised less than 1% of the population of Sherburne County in 2000, they comprised 32% of the incarcerated population of the county [39]. A 2002 study revealed that the Sherburne County Sheriff’s office reported traffic stops of Black drivers occurred five times more often than expected based on vehicle availability [44]. The study also found that while only 15% of traffic stops of White drivers were for reasons other than a driving violation, 27% of traffic stops of Black drivers were [44]. According to a ProPublica analysis of statistics from the Becker Public School District in Shelburne County for the 2014–2015 school year, Black students were 7.2 times more likely to be suspended than White students [41]. Although Black students made up only 1% of the student population in the district, they comprised 30% of students who were suspended [41]. In the Big Lake Public School District, Black students were 6.3 times more likely to be suspended than White students [41]. In the Elk River Public School District, Black students were 7.8 times more likely to be suspended than White students and on average were 1.3 grades behind White students academically [41]. As recently as 2022, the Becker school board was considering a new policy that would “restrict students’ freedom to learn complete lessons in American history, including the role of racism [45].” The board rescinded the policy only after being threatened with a lawsuit. The proposed policy elicited a letter from the ACLU, stating: “Our country needs to acknowledge and reckon with its history of systemic racism, misogyny, and discrimination against LGBTQ people — this includes being able to teach and talk about these concepts in our schools. And chilling conversations about race — and gender and sexuality — in schools risks maintaining or creating education environments that are unwelcoming to students of color, women and girls, and LGBTQ + students [46].”

Henry County, Illinois, had approximately 49,284 residents in 2020, of whom 1029 (2.1%) identified as non-Hispanic Black. Perhaps the most striking indicators we observed for Henry County were its high level or racial segregation (index of dissimilarity of 83) and its astounding racial disparities in the poverty rate, homeownership, and educational attainment. The White poverty rate is only 9.5%, while the Black poverty rate is 36.2%, a fourfold disparity. While only 19% of White residents of Henry County live in rental housing, 81% of Black residents do, a fourfold disparity. While only 6% of White adults in Henry County lack a high school education, 36% of Black residents do, a sixfold disparity. There is also a 30-fold disparity in the jailing rate. According to a ProPublica analysis of statistics from the Wethersfield Community School District in Henry County for the 2014–2015 school year, Black students were 15 times more likely to be suspended than White students [41]. Although Black students made up only 1% of the student population in the district, they comprised 30% of students who were suspended [41]. There is evidence to suggest that the Ku Klux Klan was active in Henry County in the mid-1900s, as a commenter about the book “Women of the Klan: Racism and Gender in the 1920s” noted that his great aunt, a Henry County farmer (1885–1975), was a member of the Klan [47]. The state of Illinois has a long history of extensive, restrictive covenants disallowing Black people from living in certain subdivisions; it was only in 2022 that the state finally implemented a law to allow these restrictive covenants to be removed [48].

## Limitations

There are several important limitations of this research. First, while the confirmatory factor analysis is able to confirm model fit, it does not demonstrate that the latent variable we are modeling is a valid measure of structural racism. Validating measures of structural racism is difficult since there is no “gold standard” measure with which to compare. There are statistical techniques that can be used to assess construct validity, but they are beyond the scope of this paper. Second, it is important to note that although the finding of a relationship between structural racism factor scores and the racial disparity in firearm homicide rates demonstrates the utility of our derived measure, it does not validate the measure, especially its construct validity. Third, there are numerous counties with either missing data or too small of a Black population to derive stable estimates; thus, we were not able to calculate structural racism indices for all counties. Nevertheless, we were able to capture data for counties that comprise 82% of the total US population and 89% of the non-Hispanic Black US population. In addition, we were able to include a large number of rural counties. Of the 1181 counties for which we derived structural racism factor scores, 419 had a total population of less than 50,000. Still, one limitation of this research is that it is difficult or not possible to obtain stable estimates for counties with a very small Black population. Researchers might look to small area estimate techniques to improve coverage in the future.

Fourth, while most of the data were from 2020, the county data on local jailing rates was only available from the 2010 Decennial Census. These numbers should be updated once the 2020 Decennial Census data on group quarters population are released. Fifth, while our structural racism measure incorporates five domains, but there are others that might be worthy of inclusion: for example, environmental justice, political participation, credit, and media. Data limitations have so far precluded the inclusion of these domains. However, it is possible that some of them could be included at the state level because of the increased availability of state-level data. Sixth, people do not spend all of their time in one county and may, for example, commute to another county to work. They could be affected by structural racism in multiple counties, so this needs to be kept in mind when applying these measures to actual living situations. Finally, we focused on structural racism affecting the non-Hispanic, Black population only. Further research should extend structural racism measures to other racial/ethnic groups.

## Conclusion

Despite these limitations, this study advances the use of an approach that researchers can use to derive a multidimensional, yet composite scale to measure structural racism and relate it to the study of racial health disparities. The structural racism measure presented in this paper is unique in that it is just the second to provide a single, composite measure of structural racism comprising multiple domains, using empirical methods to derive appropriate weights for each domain, uses recent, publicly available data, and extends to the county level. This will help researchers better operationalize structural racism, enhancing their ability to examine structural racism as an underlying cause of racial health disparities. At the same time, it will help public health advocates to engage in a more empirical approach to promoting racial justice policies.

## Data Availability

The database of structural racism factor scores produced in this research project are available upon request from the lead author.
